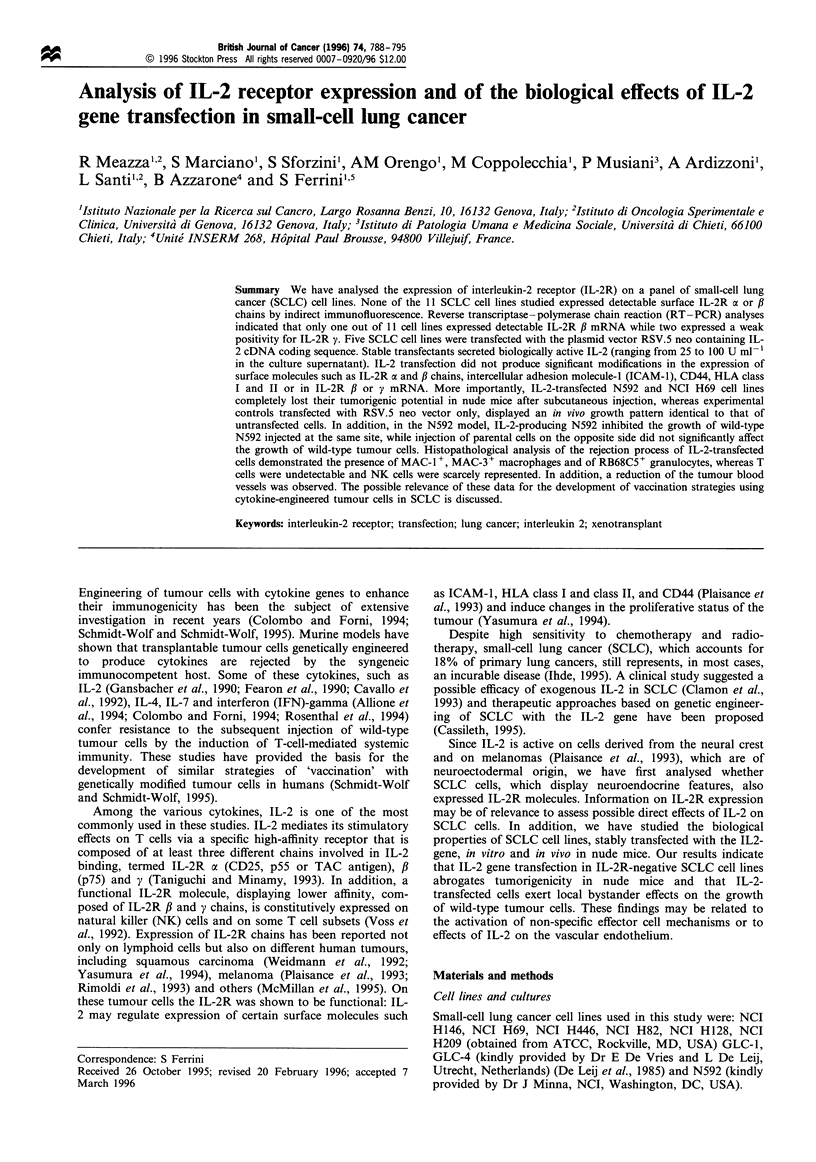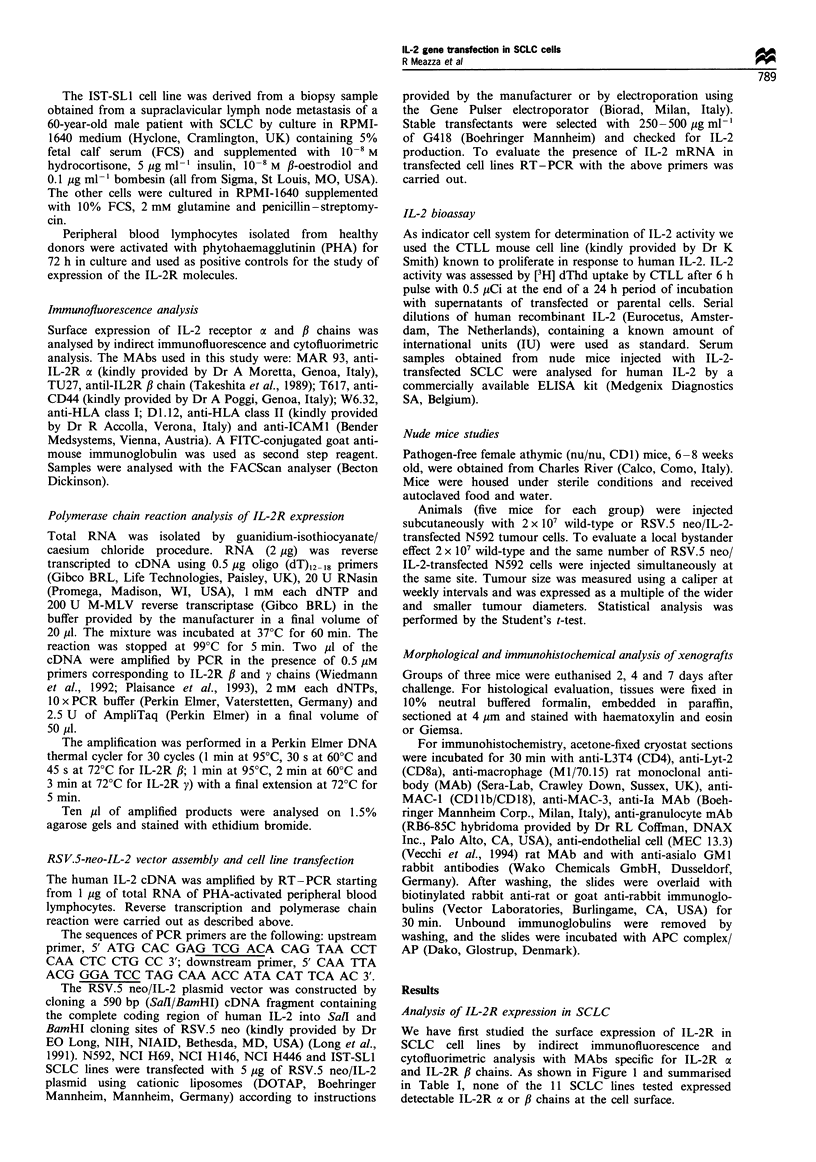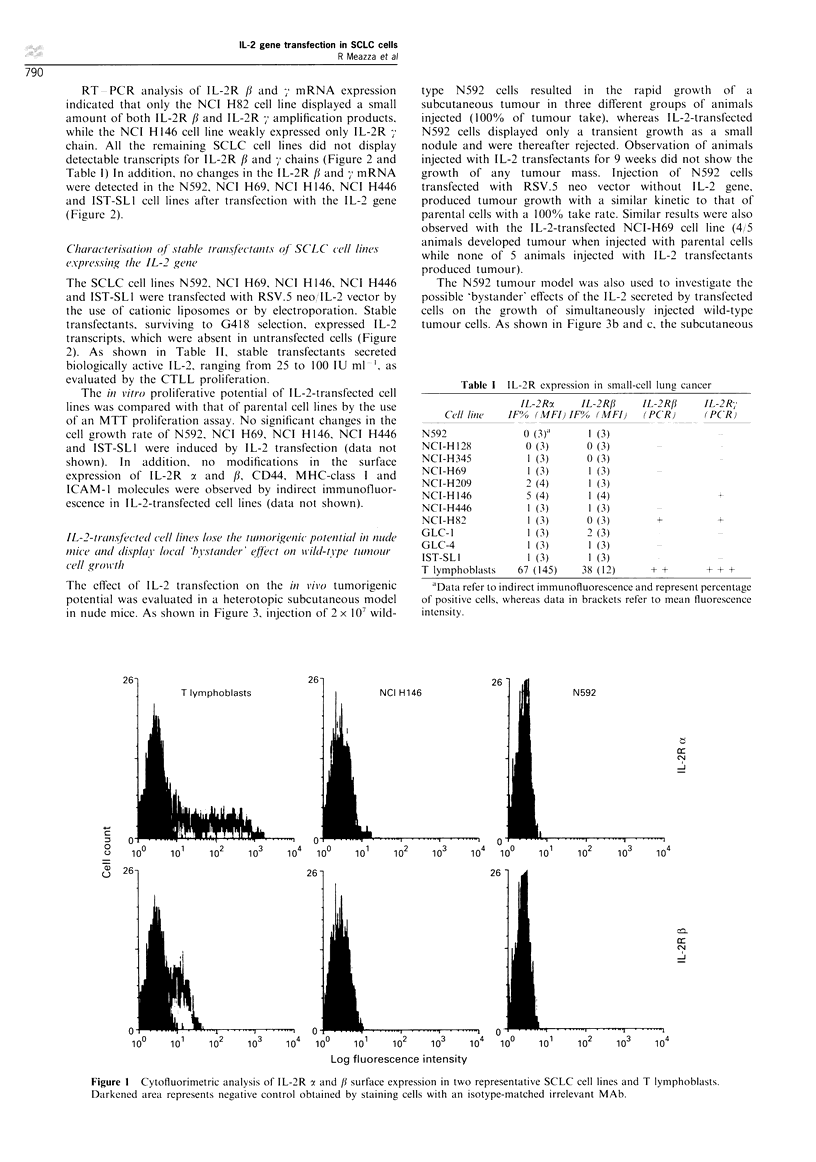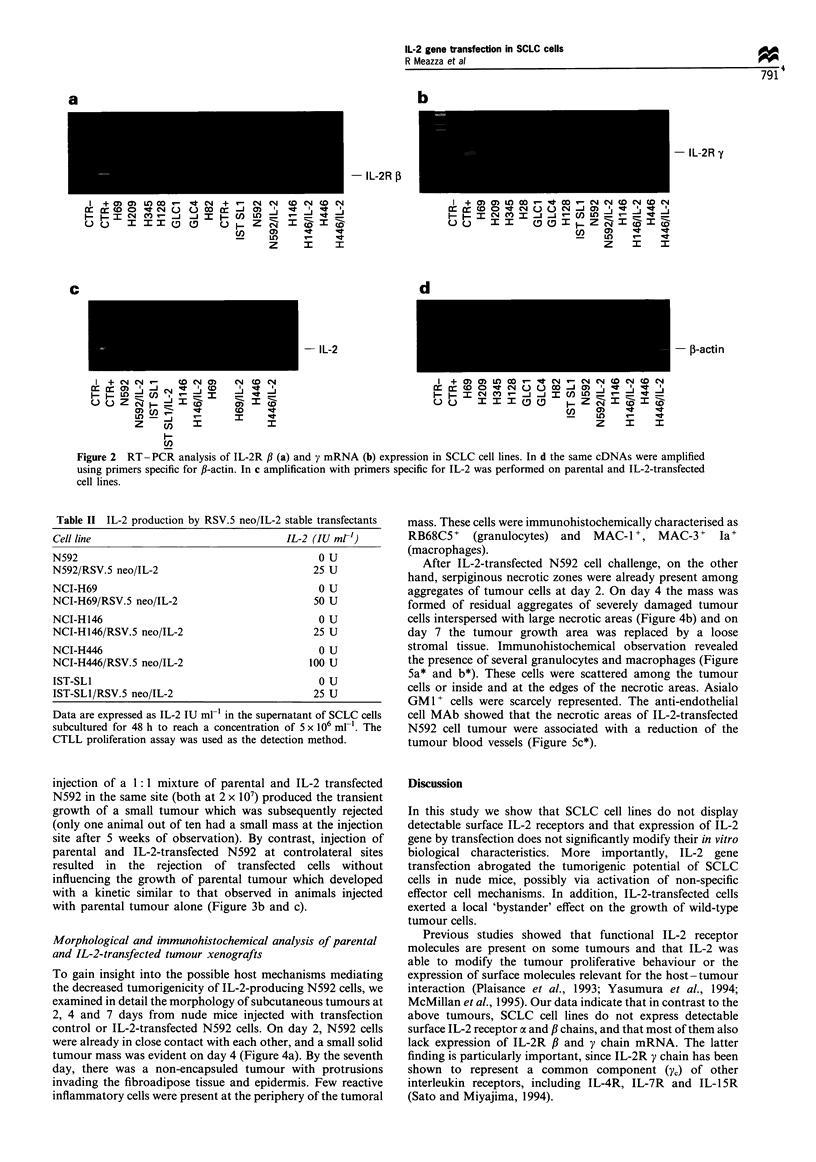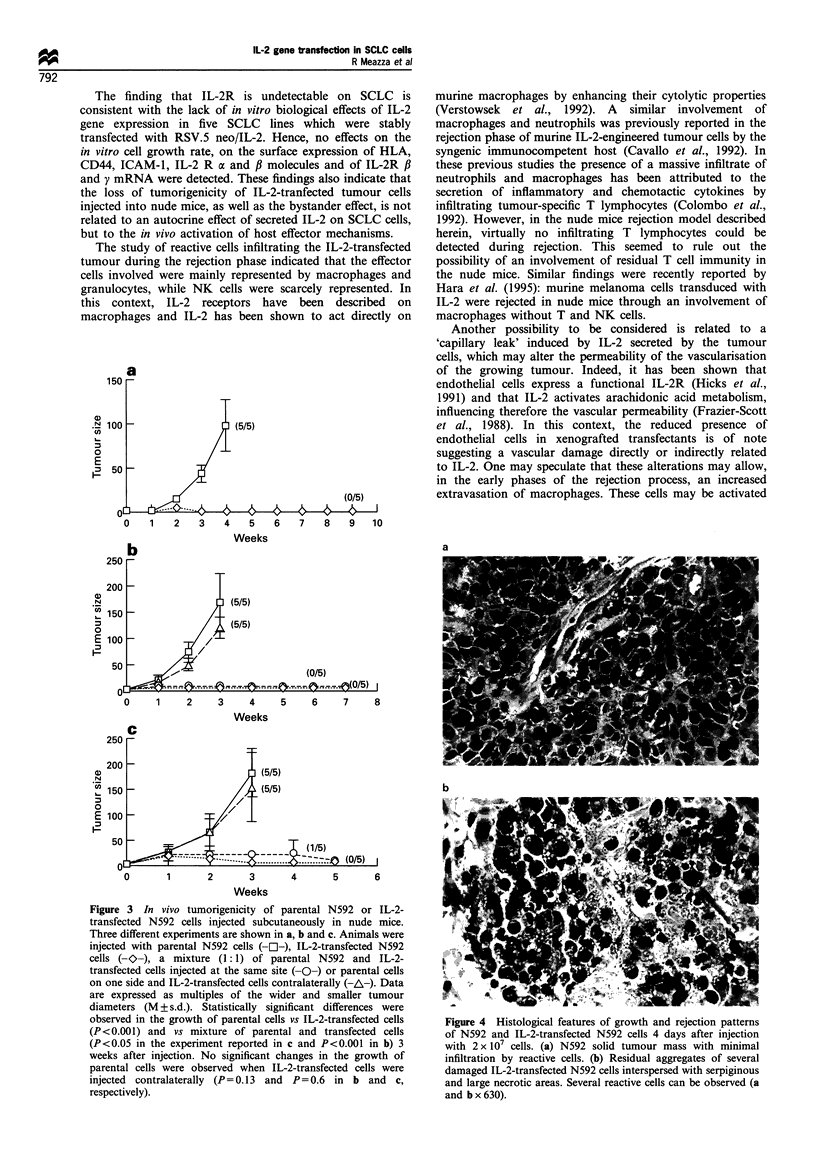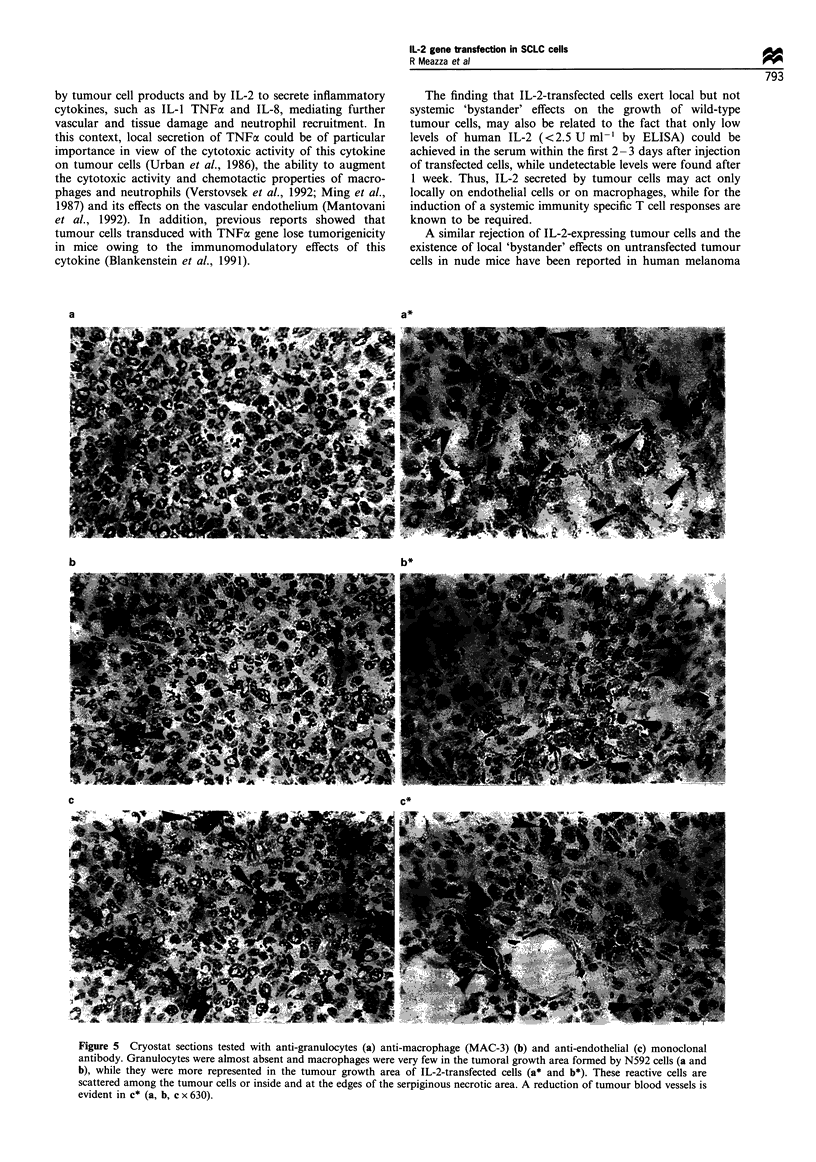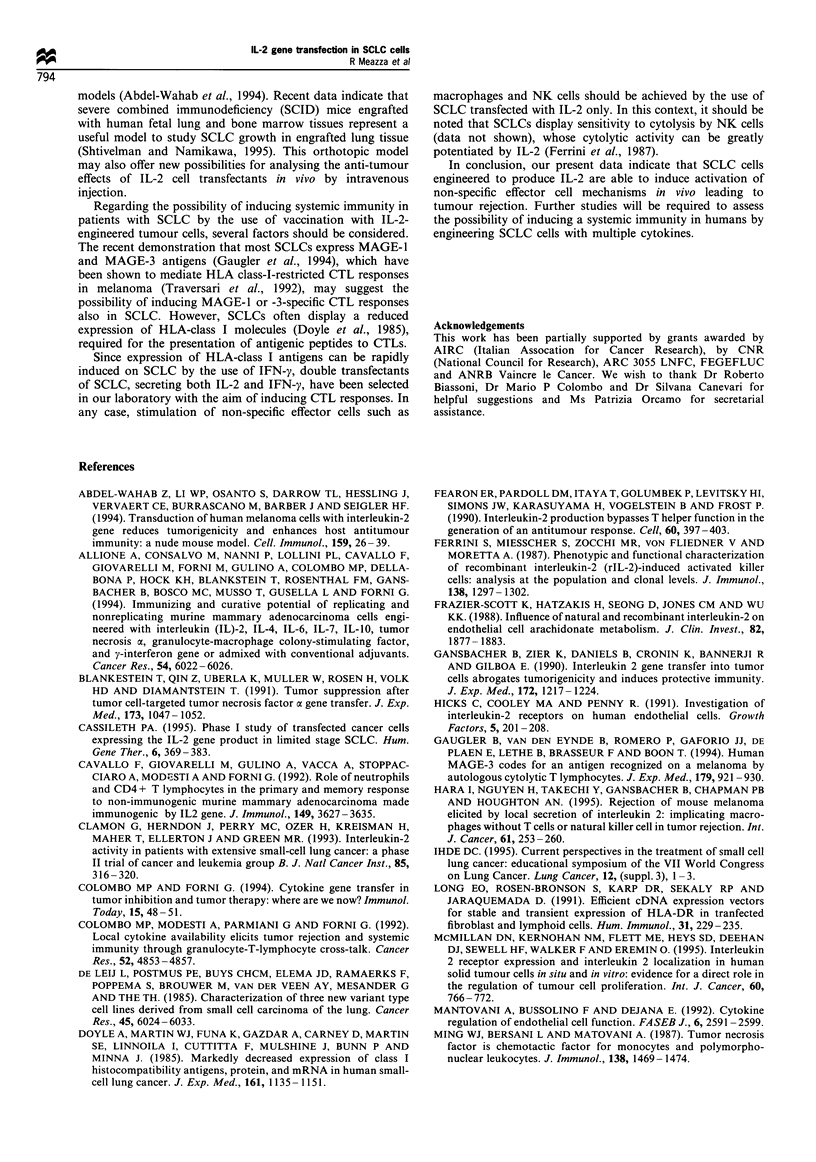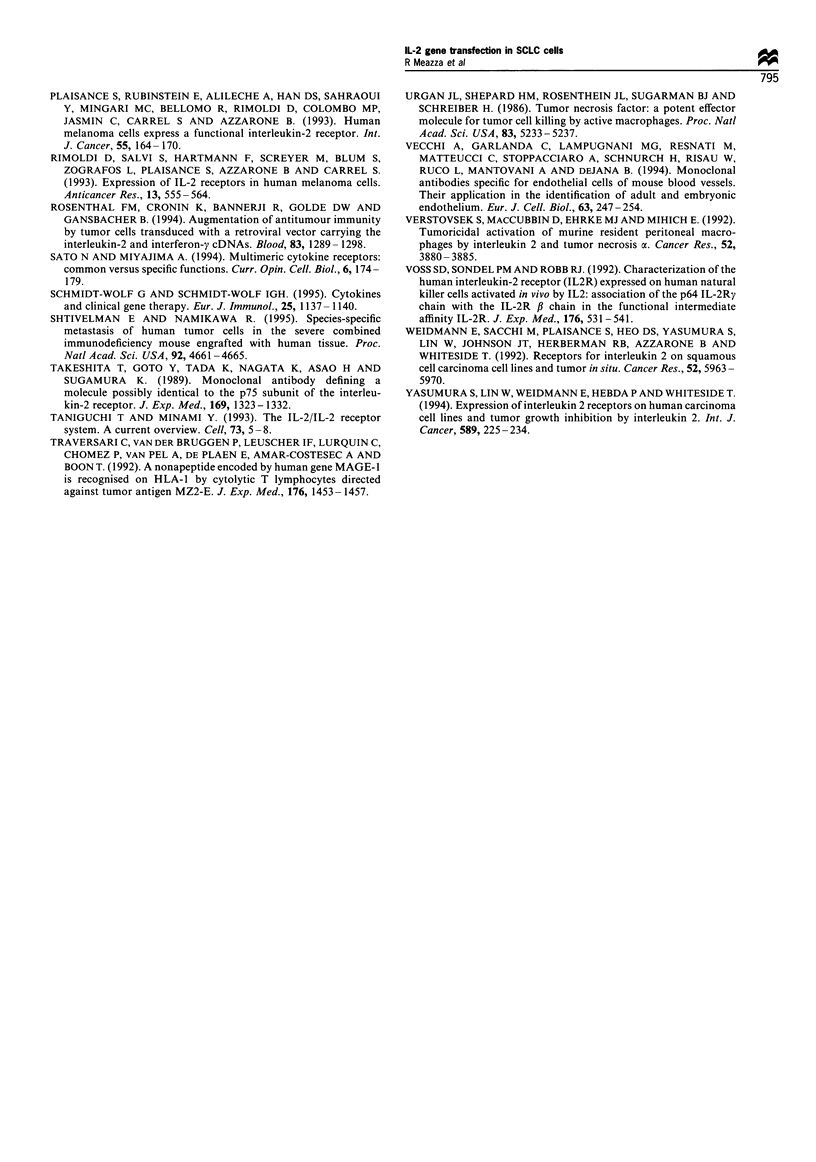# Analysis of IL-2 receptor expression and of the biological effects of IL-2 gene transfection in small-cell lung cancer.

**DOI:** 10.1038/bjc.1996.437

**Published:** 1996-09

**Authors:** R. Meazza, S. Marciano, S. Sforzini, A. M. Orengo, M. Coppolecchia, P. Musiani, A. Ardizzoni, L. Santi, B. Azzarone, S. Ferrini

**Affiliations:** Istituto Nazionale per la Ricerca sul Cancro, Genova, Italy.

## Abstract

**Images:**


British Journal of Cancer (1996) 74, 788-795
© 1996 Stockton Press All rights reserved 0007-0920/96 $12.00

### Analysis of IL-2 receptor expression and of the biological effects of IL-2 gene transfection in small-cell lung cancer

R Meazza[1,2], S Marciano[1], S Sforzini[1], AM Orengo[1], M Coppolecchia[1], P Musiani[3], A Ardizzoni[1], L Santi[1,2], B Azzarone[4] and S Ferrini[1,5]

[1]*Istituto Nazionale per la Ricerca sul Cancro, Largo Rosanna Benzi, 10, 16132 Genova, Italy;* [2]*Istituto di Oncologia Sperimentale e Clinica, Università di Genova, 16132 Genova, Italy;* [3]*Istituto di Patologia Umana e Medicina Sociale, Università di Chieti, 66100 Chieti, Italy;* [4]*Unité INSERM 268, Hôpital Paul Brousse, 94800 Villejuif, France.*

**Summary** We have analysed the expression of interleukin-2 receptor (IL-2R) on a panel of small-cell lung cancer (SCLC) cell lines. None of the 11 SCLC cell lines studied expressed detectable surface IL-2R $\alpha$ or $\beta$ chains by indirect immunofluorescence. Reverse transcriptase–polymerase chain reaction (RT–PCR) analyses indicated that only one out of 11 cell lines expressed detectable IL-2R $\beta$ mRNA while two expressed a weak positivity for IL-2R $\gamma$. Five SCLC cell lines were transfected with the plasmid vector RSV.5 neo containing IL-2 cDNA coding sequence. Stable transfectants secreted biologically active IL-2 (ranging from 25 to 100 U $ml^{-1}$ in the culture supernatant). IL-2 transfection did not produce significant modifications in the expression of surface molecules such as IL-2R $\alpha$ and $\beta$ chains, intercellular adhesion molecule-1 (ICAM-1), CD44, HLA class I and II or in IL-2R $\beta$ or $\gamma$ mRNA. More importantly, IL-2-transfected N592 and NCI H69 cell lines completely lost their tumorigenic potential in nude mice after subcutaneous injection, whereas experimental controls transfected with RSV.5 neo vector only, displayed an *in vivo* growth pattern identical to that of untransfected cells. In addition, in the N592 model, IL-2-producing N592 inhibited the growth of wild-type N592 injected at the same site, while injection of parental cells on the opposite side did not significantly affect the growth of wild-type tumour cells. Histopathological analysis of the rejection process of IL-2-transfected cells demonstrated the presence of MAC-1$^+$, MAC-3$^+$ macrophages and of RB68C5$^+$ granulocytes, whereas T cells were undetectable and NK cells were scarcely represented. In addition, a reduction of the tumour blood vessels was observed. The possible relevance of these data for the development of vaccination strategies using cytokine-engineered tumour cells in SCLC is discussed.

**Keywords:** interleukin-2 receptor; transfection; lung cancer; interleukin 2; xenotransplant

Engineering of tumour cells with cytokine genes to enhance their immunogenicity has been the subject of extensive investigation in recent years (Colombo and Forni, 1994; Schmidt-Wolf and Schmidt-Wolf, 1995). Murine models have shown that transplantable tumour cells genetically engineered to produce cytokines are rejected by the syngeneic immunocompetent host. Some of these cytokines, such as IL-2 (Gansbacher *et al.*, 1990; Fearon *et al.*, 1990; Cavallo *et al.*, 1992), IL-4, IL-7 and interferon (IFN)-gamma (Allione *et al.*, 1994; Colombo and Forni, 1994; Rosenthal *et al.*, 1994) confer resistance to the subsequent injection of wild-type tumour cells by the induction of T-cell-mediated systemic immunity. These studies have provided the basis for the development of similar strategies of 'vaccination' with genetically modified tumour cells in humans (Schmidt-Wolf and Schmidt-Wolf, 1995).

Among the various cytokines, IL-2 is one of the most commonly used in these studies. IL-2 mediates its stimulatory effects on T cells via a specific high-affinity receptor that is composed of at least three different chains involved in IL-2 binding, termed IL-2R $\alpha$ (CD25, p55 or TAC antigen), $\beta$ (p75) and $\gamma$ (Taniguchi and Minamy, 1993). In addition, a functional IL-2R molecule, displaying lower affinity, composed of IL-2R $\beta$ and $\gamma$ chains, is constitutively expressed on natural killer (NK) cells and on some T cell subsets (Voss *et al.*, 1992). Expression of IL-2R chains has been reported not only on lymphoid cells but also on different human tumours, including squamous carcinoma (Weidmann *et al.*, 1992; Yasumura *et al.*, 1994), melanoma (Plaisance *et al.*, 1993; Rimoldi *et al.*, 1993) and others (McMillan *et al.*, 1995). On these tumour cells the IL-2R was shown to be functional: IL-2 may regulate expression of certain surface molecules such as ICAM-1, HLA class I and class II, and CD44 (Plaisance *et al.*, 1993) and induce changes in the proliferative status of the tumour (Yasumura *et al.*, 1994).

Despite high sensitivity to chemotherapy and radiotherapy, small-cell lung cancer (SCLC), which accounts for 18% of primary lung cancers, still represents, in most cases, an incurable disease (Ihde, 1995). A clinical study suggested a possible efficacy of exogenous IL-2 in SCLC (Clamon *et al.*, 1993) and therapeutic approaches based on genetic engineering of SCLC with the IL-2 gene have been proposed (Cassileth, 1995).

Since IL-2 is active on cells derived from the neural crest and on melanomas (Plaisance *et al.*, 1993), which are of neuroectodermal origin, we have first analysed whether SCLC cells, which display neuroendocrine features, also expressed IL-2R molecules. Information on IL-2R expression may be of relevance to assess possible direct effects of IL-2 on SCLC cells. In addition, we have studied the biological properties of SCLC cell lines, stably transfected with the IL2-gene, *in vitro* and *in vivo* in nude mice. Our results indicate that IL-2 gene transfection in IL-2R-negative SCLC cell lines abrogates tumorigenicity in nude mice and that IL-2-transfected cells exert local bystander effects on the growth of wild-type tumour cells. These findings may be related to the activation of non-specific effector cell mechanisms or to effects of IL-2 on the vascular endothelium.

### Materials and methods

### *Cell lines and cultures*

Small-cell lung cancer cell lines used in this study were: NCI H146, NCI H69, NCI H446, NCI H82, NCI H128, NCI H209 (obtained from ATCC, Rockville, MD, USA) GLC-1, GLC-4 (kindly provided by Dr E De Vries and L De Leij, Utrecht, Netherlands) (De Leij *et al.*, 1985) and N592 (kindly provided by Dr J Minna, NCI, Washington, DC, USA).

Correspondence: S Ferrini
Received 26 October 1995; revised 20 February 1996; accepted 7 March 1996

The IST-SL1 cell line was derived from a biopsy sample obtained from a supraclavicular lymph node metastasis of a 60-year-old male patient with SCLC by culture in RPMI-1640 medium (Hyclone, Cramlington, UK) containing 5% fetal calf serum (FCS) and supplemented with $10^{-8}$ M hydrocortisone, 5 $\mu$g ml$^{-1}$ insulin, $10^{-8}$ M $\beta$-oestrodiol and 0.1 $\mu$g ml$^{-1}$ bombesin (all from Sigma, St Louis, MO, USA). The other cells were cultured in RPMI-1640 supplemented with 10% FCS, 2 mM glutamine and penicillin–streptomycin.

Peripheral blood lymphocytes isolated from healthy donors were activated with phytohaemagglutinin (PHA) for 72 h in culture and used as positive controls for the study of expression of the IL-2R molecules.

*Immunofluorescence analysis*

Surface expression of IL-2 receptor $\alpha$ and $\beta$ chains was analysed by indirect immunofluorescence and cytofluorimetric analysis. The MAbs used in this study were: MAR 93, anti-IL-2R $\alpha$ (kindly provided by Dr A Moretta, Genoa, Italy), TU27, antil-IL2R $\beta$ chain (Takeshita *et al.*, 1989); T617, anti-CD44 (kindly provided by Dr A Poggi, Genoa, Italy); W6.32, anti-HLA class I; D1.12, anti-HLA class II (kindly provided by Dr R Accolla, Verona, Italy) and anti-ICAM1 (Bender Medsystems, Vienna, Austria). A FITC-conjugated goat anti-mouse immunoglobulin was used as second step reagent. Samples were analysed with the FACScan analyser (Becton Dickinson).

*Polymerase chain reaction analysis of IL-2R expression*

Total RNA was isolated by guanidium-isothiocyanate/caesium chloride procedure. RNA (2 $\mu$g) was reverse transcripted to cDNA using 0.5 $\mu$g oligo $(dT)_{12-18}$ primers (Gibco BRL, Life Technologies, Paisley, UK), 20 U RNasin (Promega, Madison, WI, USA), 1 mM each dNTP and 200 U M-MLV reverse transcriptase (Gibco BRL) in the buffer provided by the manufacturer in a final volume of 20 $\mu$l. The mixture was incubated at 37°C for 60 min. The reaction was stopped at 99°C for 5 min. Two $\mu$l of the cDNA were amplified by PCR in the presence of 0.5 $\mu$M primers corresponding to IL-2R $\beta$ and $\gamma$ chains (Wiedmann *et al.*, 1992; Plaisance *et al.*, 1993), 2 mM each dNTPs, 10 × PCR buffer (Perkin Elmer, Vaterstetten, Germany) and 2.5 U of AmpliTaq (Perkin Elmer) in a final volume of 50 $\mu$l.

The amplification was performed in a Perkin Elmer DNA thermal cycler for 30 cycles (1 min at 95°C, 30 s at 60°C and 45 s at 72°C for IL-2R $\beta$; 1 min at 95°C, 2 min at 60°C and 3 min at 72°C for IL-2R $\gamma$) with a final extension at 72°C for 5 min.

Ten $\mu$l of amplified products were analysed on 1.5% agarose gels and stained with ethidium bromide.

*RSV.5-neo-IL-2 vector assembly and cell line transfection*

The human IL-2 cDNA was amplified by RT–PCR starting from 1 $\mu$g of total RNA of PHA-activated peripheral blood lymphocytes. Reverse transcription and polymerase chain reaction were carried out as described above.

The sequences of PCR primers are the following: upstream primer, 5′ ATG CAC GAG TCG ACA CAG TAA CCT CAA CTC CTG CC 3′; downstream primer, 5′ CAA TTA ACG GGA TCC TAG CAA ACC ATA CAT TCA AC 3′.

The RSV.5 neo/IL-2 plasmid vector was constructed by cloning a 590 bp (*Sal*I/*Bam*HI) cDNA fragment containing the complete coding region of human IL-2 into *Sal*I and *Bam*HI cloning sites of RSV.5 neo (kindly provided by Dr EO Long, NIH, NIAID, Bethesda, MD, USA) (Long *et al.*, 1991). N592, NCI H69, NCI H146, NCI H446 and IST-SL1 SCLC lines were transfected with 5 $\mu$g of RSV.5 neo/IL-2 plasmid using cationic liposomes (DOTAP, Boehringer Mannheim, Mannheim, Germany) according to instructions provided by the manufacturer or by electroporation using the Gene Pulser electroporator (Biorad, Milan, Italy). Stable transfectants were selected with 250–500 $\mu$g ml$^{-1}$ of G418 (Boehringer Mannheim) and checked for IL-2 production. To evaluate the presence of IL-2 mRNA in transfected cell lines RT–PCR with the above primers was carried out.

*IL-2 bioassay*

As indicator cell system for determination of IL-2 activity we used the CTLL mouse cell line (kindly provided by Dr K Smith) known to proliferate in response to human IL-2. IL-2 activity was assessed by [$^3$H] dThd uptake by CTLL after 6 h pulse with 0.5 $\mu$Ci at the end of a 24 h period of incubation with supernatants of transfected or parental cells. Serial dilutions of human recombinant IL-2 (Eurocetus, Amsterdam, The Netherlands), containing a known amount of international units (IU) were used as standard. Serum samples obtained from nude mice injected with IL-2-transfected SCLC were analysed for human IL-2 by a commercially available ELISA kit (Medgenix Diagnostics SA, Belgium).

*Nude mice studies*

Pathogen-free female athymic (nu/nu, CD1) mice, 6–8 weeks old, were obtained from Charles River (Calco, Como, Italy). Mice were housed under sterile conditions and received autoclaved food and water.

Animals (five mice for each group) were injected subcutaneously with $2 \times 10^7$ wild-type or RSV.5 neo/IL-2-transfected N592 tumour cells. To evaluate a local bystander effect $2 \times 10^7$ wild-type and the same number of RSV.5 neo/IL-2-transfected N592 cells were injected simultaneously at the same site. Tumour size was measured using a caliper at weekly intervals and was expressed as a multiple of the wider and smaller tumour diameters. Statistical analysis was performed by the Student's *t*-test.

*Morphological and immunohistochemical analysis of xenografts*

Groups of three mice were euthanised 2, 4 and 7 days after challenge. For histological evaluation, tissues were fixed in 10% neutral buffered formalin, embedded in paraffin, sectioned at 4 $\mu$m and stained with haematoxylin and eosin or Giemsa.

For immunohistochemistry, acetone-fixed cryostat sections were incubated for 30 min with anti-L3T4 (CD4), anti-Lyt-2 (CD8a), anti-macrophage (M1/70.15) rat monoclonal antibody (MAb) (Sera-Lab, Crawley Down, Sussex, UK), anti-MAC-1 (CD11b/CD18), anti-MAC-3, anti-Ia MAb (Boehringer Mannheim Corp., Milan, Italy), anti-granulocyte mAb (RB6-85C hybridoma provided by Dr RL Coffman, DNAX Inc., Palo Alto, CA, USA), anti-endothelial cell (MEC 13.3) (Vecchi *et al.*, 1994) rat MAb and with anti-asialo GM1 rabbit antibodies (Wako Chemicals GmbH, Dusseldorf, Germany). After washing, the slides were overlaid with biotinylated rabbit anti-rat or goat anti-rabbit immunoglobulins (Vector Laboratories, Burlingame, CA, USA) for 30 min. Unbound immunoglobulins were removed by washing, and the slides were incubated with APC complex/AP (Dako, Glostrup, Denmark).

### Results

*Analysis of IL-2R expression in SCLC*

We have first studied the surface expression of IL-2R in SCLC cell lines by indirect immunofluorescence and cytofluorimetric analysis with MAbs specific for IL-2R $\alpha$ and IL-2R $\beta$ chains. As shown in Figure 1 and summarised in Table I, none of the 11 SCLC lines tested expressed detectable IL-2R $\alpha$ or $\beta$ chains at the cell surface.

RT–PCR analysis of IL-2R $\beta$ and $\gamma$ mRNA expression indicated that only the NCI H82 cell line displayed a small amount of both IL-2R $\beta$ and IL-2R $\gamma$ amplification products, while the NCI H146 cell line weakly expressed only IL-2R $\gamma$ chain. All the remaining SCLC cell lines did not display detectable transcripts for IL-2R $\beta$ and $\gamma$ chains (Figure 2 and Table I) In addition, no changes in the IL-2R $\beta$ and $\gamma$ mRNA were detected in the N592, NCI H69, NCI H146, NCI H446 and IST-SL1 cell lines after transfection with the IL-2 gene (Figure 2).

### *Characterisation of stable transfectants of SCLC cell lines expressing the IL-2 gene*

The SCLC cell lines N592, NCI H69, NCI H146, NCI H446 and IST-SL1 were transfected with RSV.5 neo/IL-2 vector by the use of cationic liposomes or by electroporation. Stable transfectants, surviving to G418 selection, expressed IL-2 transcripts, which were absent in untransfected cells (Figure 2). As shown in Table II, stable transfectants secreted biologically active IL-2, ranging from 25 to 100 IU $ml^{-1}$, as evaluated by the CTLL proliferation.

The *in vitro* proliferative potential of IL-2-transfected cell lines was compared with that of parental cell lines by the use of an MTT proliferation assay. No significant changes in the cell growth rate of N592, NCI H69, NCI H146, NCI H446 and IST-SL1 were induced by IL-2 transfection (data not shown). In addition, no modifications in the surface expression of IL-2R $\alpha$ and $\beta$, CD44, MHC-class I and ICAM-1 molecules were observed by indirect immunofluorescence in IL-2-transfected cell lines (data not shown).

### *IL-2-transfected cell lines lose the tumorigenic potential in nude mice and display local 'bystander' effect on wild-type tumour cell growth*

The effect of IL-2 transfection on the *in vivo* tumorigenic potential was evaluated in a heterotopic subcutaneous model in nude mice. As shown in Figure 3, injection of $2 \times 10^7$ wild-type N592 cells resulted in the rapid growth of a subcutaneous tumour in three different groups of animals injected (100% of tumour take), whereas IL-2-transfected N592 cells displayed only a transient growth as a small nodule and were thereafter rejected. Observation of animals injected with IL-2 transfectants for 9 weeks did not show the growth of any tumour mass. Injection of N592 cells transfected with RSV.5 neo vector without IL-2 gene, produced tumour growth with a similar kinetic to that of parental cells with a 100% take rate. Similar results were also observed with the IL-2-transfected NCI-H69 cell line (4/5 animals developed tumour when injected with parental cells while none of 5 animals injected with IL-2 transfectants produced tumour).

The N592 tumour model was also used to investigate the possible 'bystander' effects of the IL-2 secreted by transfected cells on the growth of simultaneously injected wild-type tumour cells. As shown in Figure 3b and c, the subcutaneous

**Table I** IL-2R expression in small-cell lung cancer

| *Cell line* | *IL-2Rα IF% (MFI)* | *IL-2Rβ IF% (MFI)* | *IL-2Rβ (PCR)* | *IL-2Rγ (PCR)* |
|---|---|---|---|---|
| N592 | 0 (3)[a] | 1 (3) | | – |
| NCI-H128 | 0 (3) | 0 (3) | – | – |
| NCI-H345 | 1 (3) | 0 (3) | | – |
| NCI-H69 | 1 (3) | 1 (3) | – | – |
| NCI-H209 | 2 (4) | 1 (3) | | |
| NCI-H146 | 5 (4) | 1 (4) | | + |
| NCI-H446 | 1 (3) | 1 (3) | – | |
| NCI-H82 | 1 (3) | 0 (3) | + | + |
| GLC-1 | 1 (3) | 2 (3) | – | – |
| GLC-4 | 1 (3) | 1 (3) | – | – |
| IST-SL1 | 1 (3) | 1 (3) | – | – |
| T lymphoblasts | 67 (145) | 38 (12) | + + | + + + |

[a]Data refer to indirect immunofluorescence and represent percentage of positive cells, whereas data in brackets refer to mean fluorescence intensity.

**Figure 1** Cytofluorimetric analysis of IL-2R $\alpha$ and $\beta$ surface expression in two representative SCLC cell lines and T lymphoblasts. Darkened area represents negative control obtained by staining cells with an isotype-matched irrelevant MAb.

**Figure 2** RT–PCR analysis of IL-2R $\beta$ (**a**) and $\gamma$ mRNA (**b**) expression in SCLC cell lines. In **d** the same cDNAs were amplified using primers specific for $\beta$-actin. In **c** amplification with primers specific for IL-2 was performed on parental and IL-2-transfected cell lines.

**Table II** IL-2 production by RSV.5 neo/IL-2 stable transfectants

| *Cell line* | *IL-2 (IU ml$^{-1}$)* |
|---|---|
| N592 | 0 U |
| N592/RSV.5 neo/IL-2 | 25 U |
| NCI-H69 | 0 U |
| NCI-H69/RSV.5 neo/IL-2 | 50 U |
| NCI-H146 | 0 U |
| NCI-H146/RSV.5 neo/IL-2 | 25 U |
| NCI-H446 | 0 U |
| NCI-H446/RSV.5 neo/IL-2 | 100 U |
| IST-SL1 | 0 U |
| IST-SL1/RSV.5 neo/IL-2 | 25 U |

Data are expressed as IL-2 IU ml$^{-1}$ in the supernatant of SCLC cells subcultured for 48 h to reach a concentration of $5 \times 10^6$ ml$^{-1}$. The CTLL proliferation assay was used as the detection method.

injection of a 1:1 mixture of parental and IL-2 transfected N592 in the same site (both at $2 \times 10^7$) produced the transient growth of a small tumour which was subsequently rejected (only one animal out of ten had a small mass at the injection site after 5 weeks of observation). By contrast, injection of parental and IL-2-transfected N592 at controlateral sites resulted in the rejection of transfected cells without influencing the growth of parental tumour which developed with a kinetic similar to that observed in animals injected with parental tumour alone (Figure 3b and c).

### *Morphological and immunohistochemical analysis of parental and IL-2-transfected tumour xenografts*

To gain insight into the possible host mechanisms mediating the decreased tumorigenicity of IL-2-producing N592 cells, we examined in detail the morphology of subcutaneous tumours at 2, 4 and 7 days from nude mice injected with transfection control or IL-2-transfected N592 cells. On day 2, N592 cells were already in close contact with each other, and a small solid tumour mass was evident on day 4 (Figure 4a). By the seventh day, there was a non-encapsuled tumour with protrusions invading the fibroadipose tissue and epidermis. Few reactive inflammatory cells were present at the periphery of the tumoral mass. These cells were immunohistochemically characterised as RB68C5$^+$ (granulocytes) and MAC-1$^+$, MAC-3$^+$ Ia$^+$ (macrophages).

After IL-2-transfected N592 cell challenge, on the other hand, serpiginous necrotic zones were already present among aggregates of tumour cells at day 2. On day 4 the mass was formed of residual aggregates of severely damaged tumour cells interspersed with large necrotic areas (Figure 4b) and on day 7 the tumour growth area was replaced by a loose stromal tissue. Immunohistochemical observation revealed the presence of several granulocytes and macrophages (Figure 5a* and b*). These cells were scattered among the tumour cells or inside and at the edges of the necrotic areas. Asialo GM1$^+$ cells were scarcely represented. The anti-endothelial cell MAb showed that the necrotic areas of IL-2-transfected N592 cell tumour were associated with a reduction of the tumour blood vessels (Figure 5c*).

### Discussion

In this study we show that SCLC cell lines do not display detectable surface IL-2 receptors and that expression of IL-2 gene by transfection does not significantly modify their *in vitro* biological characteristics. More importantly, IL-2 gene transfection abrogated the tumorigenic potential of SCLC cells in nude mice, possibly via activation of non-specific effector cell mechanisms. In addition, IL-2-transfected cells exerted a local 'bystander' effect on the growth of wild-type tumour cells.

Previous studies showed that functional IL-2 receptor molecules are present on some tumours and that IL-2 was able to modify the tumour proliferative behaviour or the expression of surface molecules relevant for the host–tumour interaction (Plaisance *et al.*, 1993; Yasumura *et al.*, 1994; McMillan *et al.*, 1995). Our data indicate that in contrast to the above tumours, SCLC cell lines do not express detectable surface IL-2 receptor $\alpha$ and $\beta$ chains, and that most of them also lack expression of IL-2R $\beta$ and $\gamma$ chain mRNA. The latter finding is particularly important, since IL-2R $\gamma$ chain has been shown to represent a common component ($\gamma_c$) of other interleukin receptors, including IL-4R, IL-7R and IL-15R (Sato and Miyajima, 1994).

The finding that IL-2R is undetectable on SCLC is consistent with the lack of *in vitro* biological effects of IL-2 gene expression in five SCLC lines which were stably transfected with RSV.5 neo/IL-2. Hence, no effects on the *in vitro* cell growth rate, on the surface expression of HLA, CD44, ICAM-1, IL-2 R $\alpha$ and $\beta$ molecules and of IL-2R $\beta$ and $\gamma$ mRNA were detected. These findings also indicate that the loss of tumorigenicity of IL-2-tranfected tumour cells injected into nude mice, as well as the bystander effect, is not related to an autocrine effect of secreted IL-2 on SCLC cells, but to the *in vivo* activation of host effector mechanisms.

The study of reactive cells infiltrating the IL-2-transfected tumour during the rejection phase indicated that the effector cells involved were mainly represented by macrophages and granulocytes, while NK cells were scarcely represented. In this context, IL-2 receptors have been described on macrophages and IL-2 has been shown to act directly on murine macrophages by enhancing their cytolytic properties (Verstowsek *et al*., 1992). A similar involvement of macrophages and neutrophils was previously reported in the rejection phase of murine IL-2-engineered tumour cells by the syngenic immunocompetent host (Cavallo *et al*., 1992). In these previous studies the presence of a massive infiltrate of neutrophils and macrophages has been attributed to the secretion of inflammatory and chemotactic cytokines by infiltrating tumour-specific T lymphocytes (Colombo *et al*., 1992). However, in the nude mice rejection model described herein, virtually no infiltrating T lymphocytes could be detected during rejection. This seemed to rule out the possibility of an involvement of residual T cell immunity in the nude mice. Similar findings were recently reported by Hara *et al.* (1995): murine melanoma cells transduced with IL-2 were rejected in nude mice through an involvement of macrophages without T and NK cells.

Another possibility to be considered is related to a 'capillary leak' induced by IL-2 secreted by the tumour cells, which may alter the permeability of the vascularisation of the growing tumour. Indeed, it has been shown that endothelial cells express a functional IL-2R (Hicks *et al*., 1991) and that IL-2 activates arachidonic acid metabolism, influencing therefore the vascular permeability (Frazier-Scott *et al*., 1988). In this context, the reduced presence of endothelial cells in xenografted transfectants is of note suggesting a vascular damage directly or indirectly related to IL-2. One may speculate that these alterations may allow, in the early phases of the rejection process, an increased extravasation of macrophages. These cells may be activated

**Figure 3** *In vivo* tumorigenicity of parental N592 or IL-2-transfected N592 cells injected subcutaneously in nude mice. Three different experiments are shown in **a**, **b** and **c**. Animals were injected with parental N592 cells (–□–), IL-2-transfected N592 cells (–◇–), a mixture (1:1) of parental N592 and IL-2-transfected cells injected at the same site (–○–) or parental cells on one side and IL-2-transfected cells contralaterally (–△–). Data are expressed as multiples of the wider and smaller tumour diameters (M$\pm$s.d.). Statistically significant differences were observed in the growth of parental cells *vs* IL-2-transfected cells ($P<0.001$) and *vs* mixture of parental and transfected cells ($P<0.05$ in the experiment reported in **c** and $P<0.001$ in **b**) 3 weeks after injection. No significant changes in the growth of parental cells were observed when IL-2-transfected cells were injected contralaterally ($P=0.13$ and $P=0.6$ in **b** and **c**, respectively).

**Figure 4** Histological features of growth and rejection patterns of N592 and IL-2-transfected N592 cells 4 days after injection with $2\times10^7$ cells. (**a**) N592 solid tumour mass with minimal infiltration by reactive cells. (**b**) Residual aggregates of several damaged IL-2-transfected N592 cells interspersed with serpiginous and large necrotic areas. Several reactive cells can be observed (**a** and **b** $\times$630).

by tumour cell products and by IL-2 to secrete inflammatory cytokines, such as IL-1 TNFα and IL-8, mediating further vascular and tissue damage and neutrophil recruitment. In this context, local secretion of TNFα could be of particular importance in view of the cytotoxic activity of this cytokine on tumour cells (Urban *et al.*, 1986), the ability to augment the cytotoxic activity and chemotactic properties of macrophages and neutrophils (Verstovsek *et al.*, 1992; Ming *et al.*, 1987) and its effects on the vascular endothelium (Mantovani *et al.*, 1992). In addition, previous reports showed that tumour cells transduced with TNFα gene lose tumorigenicity in mice owing to the immunomodulatory effects of this cytokine (Blankenstein *et al.*, 1991).

The finding that IL-2-transfected cells exert local but not systemic 'bystander' effects on the growth of wild-type tumour cells, may also be related to the fact that only low levels of human IL-2 ($<2.5$ U $ml^{-1}$ by ELISA) could be achieved in the serum within the first 2–3 days after injection of transfected cells, while undetectable levels were found after 1 week. Thus, IL-2 secreted by tumour cells may act only locally on endothelial cells or on macrophages, while for the induction of a systemic immunity specific T cell responses are known to be required.

A similar rejection of IL-2-expressing tumour cells and the existence of local 'bystander' effects on untransfected tumour cells in nude mice have been reported in human melanoma

**Figure 5** Cryostat sections tested with anti-granulocytes (**a**) anti-macrophage (MAC-3) (**b**) and anti-endothelial (**c**) monoclonal antibody. Granulocytes were almost absent and macrophages were very few in the tumoral growth area formed by N592 cells (**a** and **b**), while they were more represented in the tumour growth area of IL-2-transfected cells (**a*** and **b***). These reactive cells are scattered among the tumour cells or inside and at the edges of the serpiginous necrotic area. A reduction of tumour blood vessels is evident in **c*** (**a**, **b**, **c** × 630).

models (Abdel-Wahab *et al.*, 1994). Recent data indicate that severe combined immunodeficiency (SCID) mice engrafted with human fetal lung and bone marrow tissues represent a useful model to study SCLC growth in engrafted lung tissue (Shtivelman and Namikawa, 1995). This orthotopic model may also offer new possibilities for analysing the anti-tumour effects of IL-2 cell transfectants *in vivo* by intravenous injection.

Regarding the possibility of inducing systemic immunity in patients with SCLC by the use of vaccination with IL-2-engineered tumour cells, several factors should be considered. The recent demonstration that most SCLCs express MAGE-1 and MAGE-3 antigens (Gaugler *et al.*, 1994), which have been shown to mediate HLA class-I-restricted CTL responses in melanoma (Traversari *et al.*, 1992), may suggest the possibility of inducing MAGE-1 or -3-specific CTL responses also in SCLC. However, SCLCs often display a reduced expression of HLA-class I molecules (Doyle *et al.*, 1985), required for the presentation of antigenic peptides to CTLs.

Since expression of HLA-class I antigens can be rapidly induced on SCLC by the use of IFN-γ, double transfectants of SCLC, secreting both IL-2 and IFN-γ, have been selected in our laboratory with the aim of inducing CTL responses. In any case, stimulation of non-specific effector cells such as macrophages and NK cells should be achieved by the use of SCLC transfected with IL-2 only. In this context, it should be noted that SCLCs display sensitivity to cytolysis by NK cells (data not shown), whose cytolytic activity can be greatly potentiated by IL-2 (Ferrini *et al.*, 1987).

In conclusion, our present data indicate that SCLC cells engineered to produce IL-2 are able to induce activation of non-specific effector cell mechanisms *in vivo* leading to tumour rejection. Further studies will be required to assess the possibility of inducing a systemic immunity in humans by engineering SCLC cells with multiple cytokines.

**Acknowledgements**
This work has been partially supported by grants awarded by AIRC (Italian Assocation for Cancer Research), by CNR (National Council for Research), ARC 3055 LNFC, FEGEFLUC and ANRB Vaincre le Cancer. We wish to thank Dr Roberto Biassoni, Dr Mario P Colombo and Dr Silvana Canevari for helpful suggestions and Ms Patrizia Orcamo for secretarial assistance.

### References

ABDEL-WAHAB Z, LI WP, OSANTO S, DARROW TL, HESSLING J, VERVAERT CE, BURRASCANO M, BARBER J AND SEIGLER HF. (1994). Transduction of human melanoma cells with interleukin-2 gene reduces tumorigenicity and enhances host antitumour immunity: a nude mouse model. *Cell. Immunol.*, **159**, 26–39.

ALLIONE A, CONSALVO M, NANNI P, LOLLINI PL, CAVALLO F, GIOVARELLI M, FORNI M, GULINO A, COLOMBO MP, DELLABONA P, HOCK KH, BLANKSTEIN T, ROSENTHAL FM, GANSBACHER B, BOSCO MC, MUSSO T, GUSELLA L AND FORNI G. (1994). Immunizing and curative potential of replicating and nonreplicating murine mammary adenocarcinoma cells engineered with interleukin (IL)-2, IL-4, IL-6, IL-7, IL-10, tumor necrosis α, granulocyte-macrophage colony-stimulating factor, and γ-interferon gene or admixed with conventional adjuvants. *Cancer Res.*, **54**, 6022–6026.

BLANKESTEIN T, QIN Z, UBERLA K, MULLER W, ROSEN H, VOLK HD AND DIAMANTSTEIN T. (1991). Tumor suppression after tumor cell-targeted tumor necrosis factor α gene transfer. *J. Exp. Med.*, **173**, 1047–1052.

CASSILETH PA. (1995). Phase I study of transfected cancer cells expressing the IL-2 gene product in limited stage SCLC. *Hum. Gene Ther.*, **6**, 369–383.

CAVALLO F, GIOVARELLI M, GULINO A, VACCA A, STOPPACCIARO A, MODESTI A AND FORNI G. (1992). Role of neutrophils and CD4+ T lymphocytes in the primary and memory response to non-immunogenic murine mammary adenocarcinoma made immunogenic by IL2 gene. *J. Immunol.*, **149**, 3627–3635.

CLAMON G, HERNDON J, PERRY MC, OZER H, KREISMAN H, MAHER T, ELLERTON J AND GREEN MR. (1993). Interleukin-2 activity in patients with extensive small-cell lung cancer: a phase II trial of cancer and leukemia group *B. J. Natl Cancer Inst.*, **85**, 316–320.

COLOMBO MP AND FORNI G. (1994). Cytokine gene transfer in tumor inhibition and tumor therapy: where are we now? *Immunol. Today*, **15**, 48–51.

COLOMBO MP, MODESTI A, PARMIANI G AND FORNI G. (1992). Local cytokine availability elicits tumor rejection and systemic immunity through granulocyte-T-lymphocyte cross-talk. *Cancer Res.*, **52**, 4853–4857.

DE LEIJ L, POSTMUS PE, BUYS CHCM, ELEMA JD, RAMAERKS F, POPPEMA S, BROUWER M, VAN DER VEEN AY, MESANDER G AND THE TH. (1985). Characterization of three new variant type cell lines derived from small cell carcinoma of the lung. *Cancer Res.*, **45**, 6024–6033.

DOYLE A, MARTIN WJ, FUNA K, GAZDAR A, CARNEY D, MARTIN SE, LINNOILA I, CUTTITTA F, MULSHINE J, BUNN P AND MINNA J. (1985). Markedly decreased expression of class I histocompatibility antigens, protein, and mRNA in human small-cell lung cancer. *J. Exp. Med.*, **161**, 1135–1151.

FEARON ER, PARDOLL DM, ITAYA T, GOLUMBEK P, LEVITSKY HI, SIMONS JW, KARASUYAMA H, VOGELSTEIN B AND FROST P. (1990). Interleukin-2 production bypasses T helper function in the generation of an antitumour response. *Cell*, **60**, 397–403.

FERRINI S, MIESSCHER S, ZOCCHI MR, VON FLIEDNER V AND MORETTA A. (1987). Phenotypic and functional characterization of recombinant interleukin-2 (rIL-2)-induced activated killer cells: analysis at the population and clonal levels. *J. Immunol.*, **138**, 1297–1302.

FRAZIER-SCOTT K, HATZAKIS H, SEONG D, JONES CM AND WU KK. (1988). Influence of natural and recombinant interleukin-2 on endothelial cell arachidonate metabolism. *J. Clin. Invest.*, **82**, 1877–1883.

GANSBACHER B, ZIER K, DANIELS B, CRONIN K, BANNERJI R AND GILBOA E. (1990). Interleukin 2 gene transfer into tumor cells abrogates tumorigenicity and induces protective immunity. *J. Exp. Med.*, **172**, 1217–1224.

HICKS C, COOLEY MA AND PENNY R. (1991). Investigation of interleukin-2 receptors on human endothelial cells. *Growth Factors*, **5**, 201–208.

GAUGLER B, VAN DEN EYNDE B, ROMERO P, GAFORIO JJ, DE PLAEN E, LETHE B, BRASSEUR F AND BOON T. (1994). Human MAGE-3 codes for an antigen recognized on a melanoma by autologous cytolytic T lymphocytes. *J. Exp. Med.*, **179**, 921–930.

HARA I, NGUYEN H, TAKECHI Y, GANSBACHER B, CHAPMAN PB AND HOUGHTON AN. (1995). Rejection of mouse melanoma elicited by local secretion of interleukin 2: implicating macrophages without T cells or natural killer cell in tumor rejection. *Int. J. Cancer*, **61**, 253–260.

IHDE DC. (1995). Current perspectives in the treatment of small cell lung cancer: educational symposium of the VII World Congress on Lung Cancer. *Lung Cancer*, **12**, (suppl. 3), 1–3.

LONG EO, ROSEN-BRONSON S, KARP DR, SEKALY RP AND JARAQUEMADA D. (1991). Efficient cDNA expression vectors for stable and transient expression of HLA-DR in tranfected fibroblast and lymphoid cells. *Hum. Immunol.*, **31**, 229–235.

McMILLAN DN, KERNOHAN NM, FLETT ME, HEYS SD, DEEHAN DJ, SEWELL HF, WALKER F AND EREMIN O. (1995). Interleukin 2 receptor expression and interleukin 2 localization in human solid tumour cells *in situ* and *in vitro*: evidence for a direct role in the regulation of tumour cell proliferation. *Int. J. Cancer*, **60**, 766–772.

MANTOVANI A, BUSSOLINO F AND DEJANA E. (1992). Cytokine regulation of endothelial cell function. *FASEB J.*, **6**, 2591–2599.

MING WJ, BERSANI L AND MATOVANI A. (1987). Tumor necrosis factor is chemotactic factor for monocytes and polymorphonuclear leukocytes. *J. Immunol.*, **138**, 1469–1474.

PLAISANCE S, RUBINSTEIN E, ALILECHE A, HAN DS, SAHRAOUI Y, MINGARI MC, BELLOMO R, RIMOLDI D, COLOMBO MP, JASMIN C, CARREL S AND AZZARONE B. (1993). Human melanoma cells express a functional interleukin-2 receptor. *Int. J. Cancer*, **55,** 164–170.

RIMOLDI D, SALVI S, HARTMANN F, SCREYER M, BLUM S, ZOGRAFOS L, PLAISANCE S, AZZARONE B AND CARREL S. (1993). Expression of IL-2 receptors in human melanoma cells. *Anticancer Res.*, **13,** 555–564.

ROSENTHAL FM, CRONIN K, BANNERJI R, GOLDE DW AND GANSBACHER B. (1994). Augmentation of antitumour immunity by tumor cells transduced with a retroviral vector carrying the interleukin-2 and interferon-$\gamma$ cDNAs. *Blood*, **83,** 1289–1298.

SATO N AND MIYAJIMA A. (1994). Multimeric cytokine receptors: common versus specific functions. *Curr. Opin. Cell. Biol.*, **6,** 174–179.

SCHMIDT-WOLF G AND SCHMIDT-WOLF IGH. (1995). Cytokines and clinical gene therapy. *Eur. J. Immunol.*, **25,** 1137–1140.

SHTIVELMAN E AND NAMIKAWA R. (1995). Species-specific metastasis of human tumor cells in the severe combined immunodeficiency mouse engrafted with human tissue. *Proc. Natl Acad. Sci. USA*, **92,** 4661–4665.

TAKESHITA T, GOTO Y, TADA K, NAGATA K, ASAO H AND SUGAMURA K. (1989). Monoclonal antibody defining a molecule possibly identical to the p75 subunit of the interleukin-2 receptor. *J. Exp. Med.*, **169,** 1323–1332.

TANIGUCHI T AND MINAMI Y. (1993). The IL-2/IL-2 receptor system. A current overview. *Cell*, **73,** 5–8.

TRAVERSARI C, VAN DER BRUGGEN P, LEUSCHER IF, LURQUIN C, CHOMEZ P, VAN PEL A, DE PLAEN E, AMAR-COSTESEC A AND BOON T. (1992). A nonapeptide encoded by human gene MAGE-1 is recognised on HLA-1 by cytolytic T lymphocytes directed against tumor antigen MZ2-E. *J. Exp. Med.*, **176,** 1453–1457.

URGAN JL, SHEPARD HM, ROSENTHEIN JL, SUGARMAN BJ AND SCHREIBER H. (1986). Tumor necrosis factor: a potent effector molecule for tumor cell killing by active macrophages. *Proc. Natl Acad. Sci. USA*, **83,** 5233–5237.

VECCHI A, GARLANDA C, LAMPUGNANI MG, RESNATI M, MATTEUCCI C, STOPPACCIARO A, SCHNURCH H, RISAU W, RUCO L, MANTOVANI A AND DEJANA B. (1994). Monoclonal antibodies specific for endothelial cells of mouse blood vessels. Their application in the identification of adult and embryonic endothelium. *Eur. J. Cell. Biol.*, **63,** 247–254.

VERSTOVSEK S, MACCUBBIN D, EHRKE MJ AND MIHICH E. (1992). Tumoricidal activation of murine resident peritoneal macrophages by interleukin 2 and tumor necrosis $\alpha$. *Cancer Res.*, **52,** 3880–3885.

VOSS SD, SONDEL PM AND ROBB RJ. (1992). Characterization of the human interleukin-2 receptor (IL2R) expressed on human natural killer cells activated *in vivo* by IL2: association of the p64 IL-2R$\gamma$ chain with the IL-2R $\beta$ chain in the functional intermediate affinity IL-2R. *J. Exp. Med.*, **176,** 531–541.

WEIDMANN E, SACCHI M, PLAISANCE S, HEO DS, YASUMURA S, LIN W, JOHNSON JT, HERBERMAN RB, AZZARONE B AND WHITESIDE T. (1992). Receptors for interleukin 2 on squamous cell carcinoma cell lines and tumor *in situ*. *Cancer Res.*, **52,** 5963–5970.

YASUMURA S, LIN W, WEIDMANN E, HEBDA P AND WHITESIDE T. (1994). Expression of interleukin 2 receptors on human carcinoma cell lines and tumor growth inhibition by interleukin 2. *Int. J. Cancer*, **589,** 225–234.